# Nitric oxide availability is increased in contracting skeletal muscle from aged mice, but does not differentially decrease muscle superoxide

**DOI:** 10.1016/j.freeradbiomed.2014.10.505

**Published:** 2015-01

**Authors:** T. Pearson, A. McArdle, M.J. Jackson

**Affiliations:** MRC–Arthritis Research UK Centre for Integrated research into Musculoskeletal Ageing (CIMA), Department of Musculoskeletal Biology Institute of Ageing and Chronic Disease, University of Liverpool, Liverpool, L69 3GA, UK

**Keywords:** DHE, dihydroethidium, MEM, minimum essential medium Eagle, NO, nitric oxide, NOS, nitric oxide synthases, 3-NT, 3-nitrotyrosine, Prx5, peroxiredoxin 5, NO, ROS, Aging, Skeletal muscle, Fluorescent confocal microscopy

## Abstract

Reactive oxygen and nitrogen species have been implicated in the loss of skeletal muscle mass and function that occurs during aging. Nitric oxide (NO) and superoxide are generated by skeletal muscle and where these are generated in proximity their chemical reaction to form peroxynitrite can compete with the superoxide dismutation to hydrogen peroxide. Changes in NO availability may therefore theoretically modify superoxide and peroxynitrite activities in tissues, but published data are contradictory regarding aging effects on muscle NO availability. We hypothesised that an age-related increase in NO generation might increase peroxynitrite generation in muscles from old mice, leading to an increased nitration of muscle proteins and decreased superoxide availability. This was examined using fluorescent probes and an isolated fiber preparation to examine NO content and superoxide in the cytosol and mitochondria of muscle fibers from adult and old mice both at rest and following contractile activity. We also examined the 3-nitrotyrosine (3-NT) and peroxiredoxin 5 (Prx5) content of muscles from mice as markers of peroxynitrite activity. Data indicate that a substantial age-related increase in NO levels occurred in muscle fibers during contractile activity and this was associated with an increase in muscle eNOS. Muscle proteins from old mice also showed an increased 3-NT content. Inhibition of NOS indicated that NO decreased superoxide bioavailability in muscle mitochondria, although this effect was not age related. Thus increased NO in muscles of old mice was associated with an increased 3-NT content that may potentially contribute to age-related degenerative changes in skeletal muscle.

## Highlights

• We studied NO in contracting and resting muscle fibers from adult and old mice.
• Contracting fibers from old mice had greater NO than those from adult mice.
• Muscle proteins from old mice contained increased 3-nitrotyrosine residues.
• Nitric oxide synthase inhibition decreased mitochondrial superoxide in both groups.
• Increased NO in muscle of old mice increases peroxynitrite and protein nitration.

## Introduction

Aging results in a loss of physical capacity and increased frailty through a reduction in skeletal muscle mass and function (sarcopenia) [1] through a substantial reduction in muscle cross-sectional area and reduced force generation [2,3]. In man such changes result in an increased risk of a fall [4] and need for residential care [5] that can have a significant impact upon an individual׳s quality of life and personal and financial independence.

During aging, the skeletal muscles of old rodents contained increased amounts of oxidised lipid, DNA, and proteins in comparison with young or adult rodents (e.g., [6–8]). Increased superoxide generation has been implicated in the process of aging in skeletal muscle and other tissues [7,9]. Superoxide and nitric oxide (NO) are the primary radical species generated in skeletal muscle and their generation is increased during contractile activity [10–13]. Superoxide and NO are the precursors for the generation of a number of secondary species and muscle has enzymatic systems to control the cellular activities of these species. When superoxide and NO are both present, their chemical reaction to form peroxynitrite is likely and competes with the dismutation of superoxide to hydrogen peroxide by SOD [14].

Previous studies have examined the activity of ROS in skeletal muscle during aging using the nonspecific fluorescent probe DCFH [15]. These data indicated that ROS activities were increased in isolated muscle fibers from old mice at rest in comparison with fibers from adult mice, but that the increase in ROS following contractile activity normally seen in fibers from adult mice was not seen in those from old mice. Unfortunately these studies do not allow the specific species involved to be determined since the DCFH probe has been reported to be oxidised by hydrogen peroxide, NO, hydroxyl radical, and peroxynitrite [16]. In a mouse model showing an accelerated skeletal muscle aging phenotype (SOD1null mice), we have previously obtained data to indicate that peroxynitrite is formed in excess in skeletal muscle and appears to play a role in the muscle loss [17]. Detection of peroxynitrite is difficult in biological processes and is usually inferred from monitoring and modification of cellular NO and superoxide that combine to form peroxynitrite and from examining the content of 3-nitrotyrosines (3-NT) of muscle proteins [8,13,17,18], since these posttranslational modifications are usually formed following the reaction of tyrosine residues with peroxynitrite [14].

A number of studies have examined the effect of age on superoxide generation from mitochondria or mitochondrial particles extracted from muscles of different species [19–22] and concluded that aging increases mitochondrial superoxide generation, but studies of NO synthesis and bioavailability in muscle during aging have provided contradictory data. NO is generated by the activity of the nitric oxide synthases (NOS) and studies have reported an age-related increase [23] or decrease [24] in the muscle protein content of nNOS (NOS1), increased iNOS (NOS3) protein content [25,26] in rodent muscle, and an increase in both nNOS and eNOS (NOS2) in muscle from older humans [27]. This latter study also examined the bioavailability of NO in muscle and conversely found an age-related decrease in interstitial NO in the presence of the increased muscle content of the 2 NOS enzymes [27].

Our hypothesis was that that an age-related increase in NO generation might increase peroxynitrite generation in muscles from old mice, leading to an increased nitration of muscle proteins and decreased superoxide availability. The aim of this study was therefore to examine NO in muscle fibers from adult and old mice both at rest and following contractile activity and to determine the effect of any age-related changes in NO on the levels of superoxide detected in cytosol and mitochondria. We also examined 3-NT and the peroxiredoxin 5 (Prx5) content of muscles from old mice as markers of increased peroxynitrite activity.

## Materials and methods

### Mice

These studies used adult (5–7 months) and old (26–28 months) male C57Bl/6 mice. All experiments were performed in accordance with UK Home Office guidelines and under the UK Animals (Scientific Procedures) Act 1986. Mice were killed by Schedule 1 and the flexor digitorum brevis (FDB) muscle was rapidly removed (see below). The gastrocnemius muscles were also rapidly removed and snap-frozen in liquid nitrogen for future analysis.

### Isolation of single mature skeletal muscle fibers

Single fibers were isolated from the FDB muscles of mice [28]. Briefly, mice were killed and the FDB muscles were rapidly dissected. Muscles were incubated for 1.5 h at 37 °C in 0.4% (w/v) sterile type I collagenase (EC 3.4.24.3, Sigma Chemical Co., Poole, Dorset, UK) in minimum essential medium Eagle (MEM) media containing 2 mM glutamine, 50 IU penicillin, 50 μg ml^−1^ streptomycin and 10% fetal bovine serum (FBS, Sigma Chemical Co., Poole, Dorset, UK). The muscles were agitated every 30 min during the digestion period. Single myofibers were released by gentle trituration with a wide-bore pipette and fibers were washed three times in MEM media containing 10% FBS. Fibers were plated onto precooled 35-mm glass-bottomed cell culture dishes (MatTek, MA, USA) precoated with Matrigel (BD Biosciences, Oxford, UK) and were allowed to attach before adding 2 ml MEM media containing 10% FBS. Fibers were incubated for 20 h at 37 °C in a 5% CO_2_ tissue culture incubator. Fibers prepared and cultured in this manner are viable for up to 6 days in culture though in this study all fibers were used within 30 h [15]. Experiments were only performed on fibers that displayed excellent morphology and exhibited prominent cross-striations.

### Chemicals

MitoSox Red, DHE, and DAF-FM DA (Invitrogen, Paisley, UK) were all made in DMSO, vehicle equivalent to 0.0125, 0.1, and 0.2%, respectively (no effect of the vehicle was found). MEM-eagles, D-PBS, and L-NAME were from Sigma Chemical Co. MEM solution consisted of (in mM) MgSO_4_∙H_2_O 0.8, KCl 5.4, NaCl 116.4, NaH_2_PO_4_∙H_2_O 1, D-glucose 5.5, NaHCO_3_ 26.2, Hepes 10, CaCl_2_∙2H_2_O 1.9 and pH 7.4; salts were acquired from Sigma Chemical Co.

### Use of DAF-FM DA to monitor nitric oxide in isolated fibers

NO availability was examined using the NO-specific probe DAF-FM essentially as described by Pye et al. [29]. In brief, fibers were loaded by incubation in 2 ml Dulbecco’s phosphate-buffered saline (D-PBS) containing 10 µM DAF-FM DA for 30 min at 37 °C in a tissue culture incubator. Cells were washed twice with D-PBS and two further washes using MEM solution; the fibers were then maintained in 2 ml MEM solution during the experimental protocol. DAF-FM DA readily diffuses into cells and within the cytoplasm releases DAF-FM by the action of intracellular esterases. DAF-FM is essentially nonfluorescent until it is nitrosylated by products of oxidation of NO, resulting in DAF-FM triazole that exhibits about a 160-fold greater fluorescence efficiency [30].

### Use of dihydroethidium to monitor cytoplasmic superoxide in isolated fibers

Cyotoplasmic superoxide was examined using dihydroethidium (DHE, hydroethidine) as described by Pearson et al. [31]. In brief, fibers were loaded by incubation in 2 ml D-PBS containing 5 μM DHE for 20 min at 37 °C in a tissue culture incubator. Cells were then washed twice with D-PBS and two further washes using MEM. The fibers were maintained in 2 ml MEM solution during the experimental protocol. Additionally, fibers were either untreated or incubated with 100 µM L-NAME to block nitric oxide synthase for a minimum of 1 h prior to loading with DHE. These fibers were maintained in L-NAME throughout the experiment.

### Use of MitoSox Red to monitor mitochondrial superoxide in isolated fibers

Mitochondrial superoxide was examined using MitoSox red as described by Pearson et al. [31]. Fibers were loaded by incubation in 2 ml D-PBS containing 125 nM MitoSox Red for 10 min at 37 °C in a tissue culture incubator. Cells were then washed twice with D-PBS and two further washes using MEM; the fibers were maintained in 2 ml MEM during the experimental protocol. Fibers were either untreated or incubated for a minimum of 1 h in the presence of 100 µM L-NAME as described above.

### Confocal microscopy

A Nikon E-Ti inverted microscope with a motorised stage (TI-S-EJOY, Nikon) for a 35-mm petri dish was used. A C1 confocal microscope (Nikon Instruments Europe BV, Surrey, UK) comprising a diode (UV) 405 nm excitation, argon laser with 488 nm excitation, and a helium–neon laser with 543 nm excitation were used for live cell imaging. Acquisition software was EZC1 V.3.9 (12 bit). DHE and MitoSox Red were excited sequentially at 405 nm using a diode laser (25% intensity) and 488 nm (3% intensity) using an argon laser, each passing through a main dichroic and secondary beam splitter with the emission collected through a 605/15 filter to a detector. DAF-FM was excited at 488 nm and emission recorded between 515 and 30 nm. Bright-field images were acquired using the 543 nm laser to a CCD. The objective was a PlanApo VC x60A/1.2NA/0.27 mm working distance water immersion. Pinhole size was 150 µm with a 1.68 µs pixel dwell time in all cases. Regions of interest for determination of fluorescence/area were selected and quantified as previously described [15,31]. Excitation at 405 nm was used for all analyses of MitoSox Red and DHE to maximise the contribution from hydroxy-Mito-ethidium and 2-hydroxyethidium products, respectively. All experiments were performed at approximately 25 °C.

### Electrical stimulation of contraction in isolated fibers

Single muscle fibers were subjected to electrical field stimulation (Harvard Apparatus, Kent, UK) in 35-mm glass-bottomed petri dishes using platinum electrodes (Advent, Oxford, UK) in a protocol lasting a total of 60 min (7 confocal images captured every 10 min). The specific experimental profile consisted of a rest period of 10 min duration followed by electrical stimulation for 10 min by trains of 30 V bipolar square wave pulses of 2 ms in duration for 0.5 s repeated every 5 s at 50 Hz. The fibers were then maintained at rest for 20 min. A second identical stimulation period was then applied followed by a 10 min period at rest [31].

### Analysis of muscle for NOS isoenzymes, 3NT, and Prx5 by Western blotting

Gastrocnemius muscles were rapidly dissected and snap-frozen in liquid nitrogen. Muscle samples were ground to a fine powder under liquid nitrogen and approximately half the powder was resuspended in 1% (w/v) SDS that included protease and phosphatase inhibitor cocktails. Samples were sonicated three times for 5 s on ice and then centrifuged at 10,600 *g* for 10 min. The supernatant protein was quantified in duplicate using Bradford Ultra (Expedeon, Cambridgeshire, UK) and prepared in Laemmli sample buffer for separation using a 12% SDS-PAGE at 75 µg protein/sample and subsequently transferred onto a nitrocellulose membrane (Sigma). Blots were probed using monoclonal antibodies for GAPDH, peroxiredoxin V, eNOS, iNOS, nNOS (Abcam, Cambridge, UK, 1:5000, 1:500, 1:750, 1:500, and 1:500, respectively), and 3-NT (Cayman Chemicals, Ann Arbour, MI, USA, 1:750). Following probing of the blot for 3-NT, the same blot was washed and reprobed for GAPDH to determine equal sample loading. Prx5 and NOS blots were cut in half and one section was probed separately with GAPDH antibody to determine sample loading level. When using GAPDH, blocking was 3% BSA throughout whereas all other blocking was with 3–5% milk. Protein was visualised after applying specific secondary HRP-conjugated antibodies and exposure to a Supersignal west dura substrate (Pierce-Thermo, Northumberland, UK). Bands were visualised using ChemiDoc XRS (Bio-Rad, Hertfordshire, UK) and band intensities quantified by densitometry.

### Statistical analysis

Data are presented as mean ± SE, where *n* represents number of mice. Data were analysed using SPSS v. 18; analysis was done using data between time points 10 and 60 min. Data distribution was checked using KS test and was normal throughout. Data were tested by general linear models repeated measures (examining treatments; exposure to drug and/or muscle stimulation and age), one-way ANOVA or Student’s *t* test as indicated. Data were considered significant at *P* < 0.05.

## Results

### Muscle content of nitric oxide synthases

**Fig. 1:**
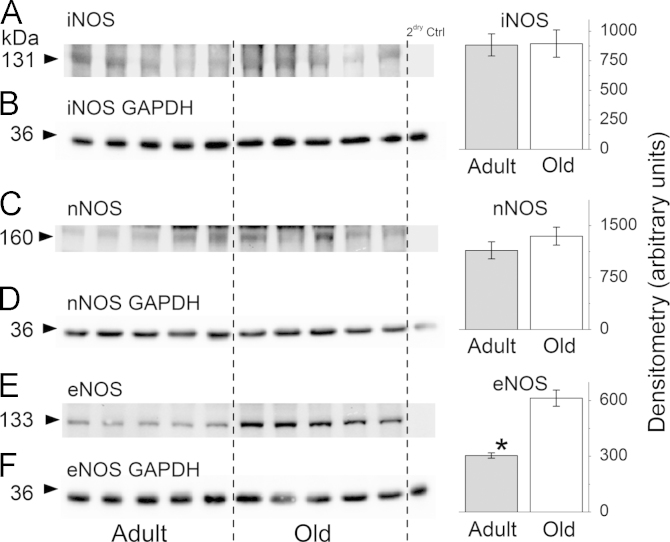
Representative Western blots and densitometric analysis of the intensity of blots for the different NOS isoforms; (A) iNOS, (C) nNOS, (E) eNOS (*n*=5 for adult and old). Blots B, D, and F show the GAPDH content as a loading control for each of the respective NOS blots. ^⁎^*P*=0.0013, significant increase in eNOS content in muscle from old mice using Student’s *t* test.

No differences were seen between the two age groups for iNOS (Fig. 1A) or nNOS protein content (Fig. 1C). A significant increase in eNOS content was found in muscle from old mice compared with the content in muscle from adult mice (Fig. 1E, Student’s *t* test, *P*=0.0013). Analysis of GAPDH as a loading control showed no differences between any group.

### Effect of age and contractile activity on nitric oxide in isolated muscle fibers

**Fig. 2:**
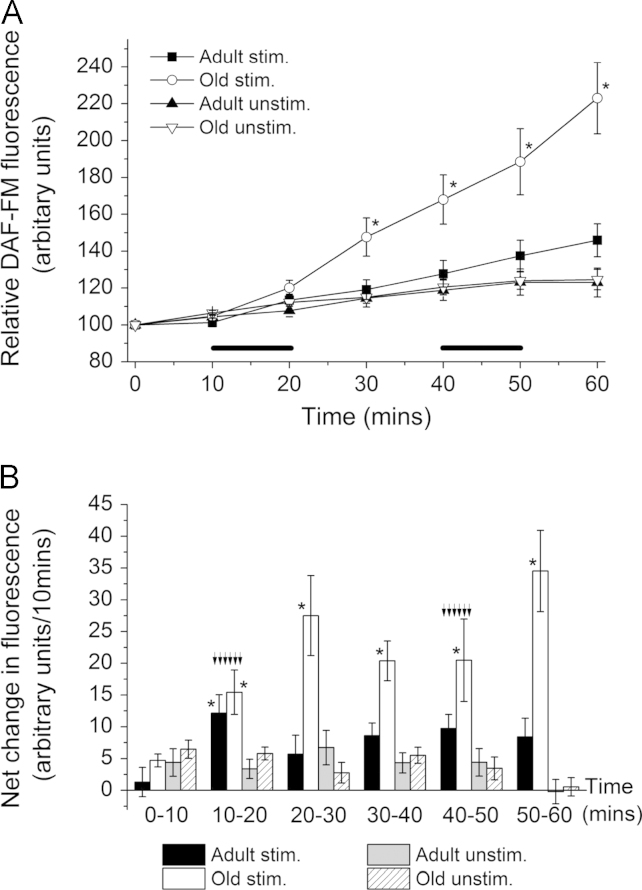
(A) Relative DAF-FM fluorescence from fibers of adult and old mice over 60 min. Fibers were either at rest (unstim.) or stimulated (stim.) to contract during two 10 min periods (denoted by the black bars, *n*=9–11 in all groups), ^⁎^*P*<0.05 compared with all other groups at the same time point using one-way ANOVA. (B) Net change in DAF-FM fluorescence/ 10 min from fibers of adult and old mice, either at rest (unstim.) or stimulated (stim.) to contract during the periods denoted by arrows. The two stimulated groups were not different from each other at 10–20 min. ^⁎^*P*<0.05, compared with all other groups at the same time point using one-way ANOVA.

Fig. 2A shows DAF-FM fluorescence from adult and old mice over 60 min, both at rest and where fibers were stimulated to contract for two 10 min periods (indicated by black bars). Analysis of the full time course showed a significant increase in fluorescence in stimulated fibers from old mice compared with unstimulated fibers (repeated measure *F*=13.6, *P*=0.002). No significant change with contractions was seen for fibers from adult mice. A comparison of contracted fibers from old mice with those from adult mice showed significantly greater fluorescence (*F*=8.6, *P*=0.009) in the fibers from old mice, indicating a greater amount of NO generation from the contracted fibers of old mice compared with those from the contracted fibers of adult mice. No differences were found between DAF-FM fluorescence in fibers from old and adult mice at rest. Overall an effect of aging was found that interacted significantly with stimulation (*F*=6.2, *P*=0.018).

Fig. 2B shows the rate of change in DAF-FM fluorescence from fibers of adult and old mice over each 10 min sampling period in response to either no stimulation or the two periods of contraction (indicated by arrows). During the first contraction period fibers from both adult and old mice showed a higher rate of increase in fluorescence compared with fibers at rest, but subsequently the fibers from the old mice showed significantly higher rates of increase in fluorescence in each 10 min period throughout the remainder of the experiment (one-way ANOVA, *P*<0.05).

### Effect of age and contractile activity on cytoplasmic and mitochondrial superoxide

**Fig. 3:**
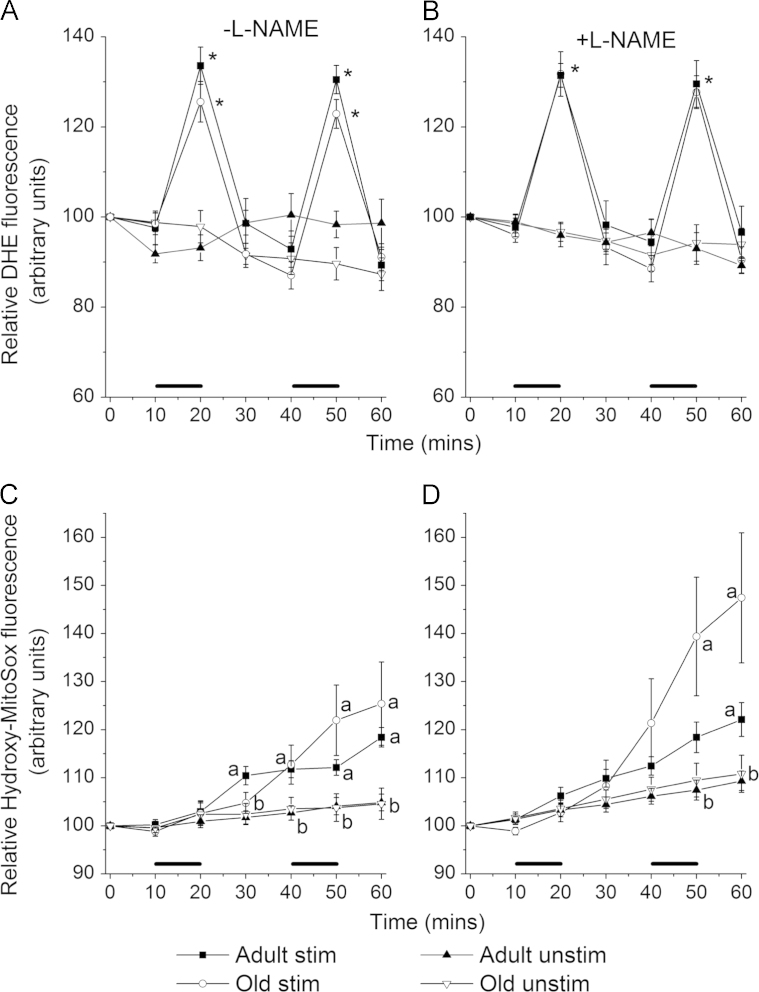
(A) Relative 2-hydroxyethidium (DHE) fluorescence from fibers of adult and old mice over 60 min. Fibers were either at rest (unstim.) or stimulated (stim.) to contract during two 10 min periods (denoted by the black bars, *n* =7–10 in all groups). ^⁎^*P*<0.05 compared with all unstimulated groups at the same time point using one-way ANOVA. (B) Repeat of experiment shown in (A) in the presence of 100 µM L-NAME to block nitric oxide generation (*n*=8–10 for all groups) ^⁎^*P*<0.05 compared with all other groups at the same time point using one-way ANOVA. (C) Relative hydroxy-Mito-ethidium fluorescence over 60 min from fibers of adult and old mice. Fibers were either at rest (unstim.) or stimulated (stim.) to contract during two 10 min periods (denoted by the black bars, *n* =8–11 for all groups), where a is significantly greater than b using one-way ANOVA, *P*<0.05. (D) Repeat of experiment shown in (C) but in the presence of 100 µM L-NAME (*n*=10–12 for all groups), where a is significantly greater than b using one-way ANOVA, *P*<0.05.

Fig. 3A shows hydroxyethidium fluorescence from muscle fibers from adult and old mice at rest and subjected to two 10 min periods of contractile activity. Fig. 3B shows the data from an identical experimental design in which the fibers were maintained in the presence of 100 µM L-NAME to block the activity of nitric oxide synthases. The pattern of superoxide generation in response to muscle contractions was similar between fibers from the mice of differing ages and both groups showed similar rapid increases in hydroxyethidium.

Analysis of the whole time course (Fig. 3A) showed significant differences between stimulated and unstimulated fibers from adult mice (repeated measures, *F*=6.4, *P*=0.026) and a similar pattern was found for fibers from old mice (*F*=9.5, *P*=0.007). Similar changes were seen in the fibers incubated in L-NAME (Fig. 3B) from adult (*F*=8.8, *P*=0.01) and old (*F*=5.8, *P*=0.027) mice. These data show a significant increase in cytoplasmic superoxide generation in response to muscle contraction in both age groups irrespective of the presence of nitric oxide. No significant differences were found between fluorescence from fibers of either adult or old mice at rest or stimulated to contract whether or not they were in the presence of L-NAME.

Fig. 3C and D show changes in hydroxy-Mito-ethidium fluorescence from fibers of adult and old mice in the absence and presence of L-NAME either stimulated to contract or unstimulated. In the absence of L-NAME muscle contractions caused an overall increase in hydroxy-Mito-ethidium fluorescence in adult (*F*=15.8, *P*=0.001) fibers compared with unstimulated fibers, although fibers from old mice just failed to attain significance (*F*=4.2, *P*=0.054). In the presence of L-NAME (Fig. 3D) there was also a significant increase in hydroxy-Mito-ethidium fluorescence in response to muscle contraction in both fibers from adult and old mice. L-NAME appeared to induce an increase in fluorescence from fibers in all groups, which was greatest in the contracted fibers from old mice but this failed to achieve statistical significance in either age group when comparing the fibers in the presence of L-NAME to those where it was not present. Global analysis between all data from fibers of adult and old mice showed a significant effect of contraction (*F*=18.6, *P*=0.001) and of L-NAME (*F*=4.8, *P*=0.031) over the whole time course.

### Effect of age on the 3-nitrotyrosine and peroxiredoxin V contents of muscle.

**Fig. 4:**
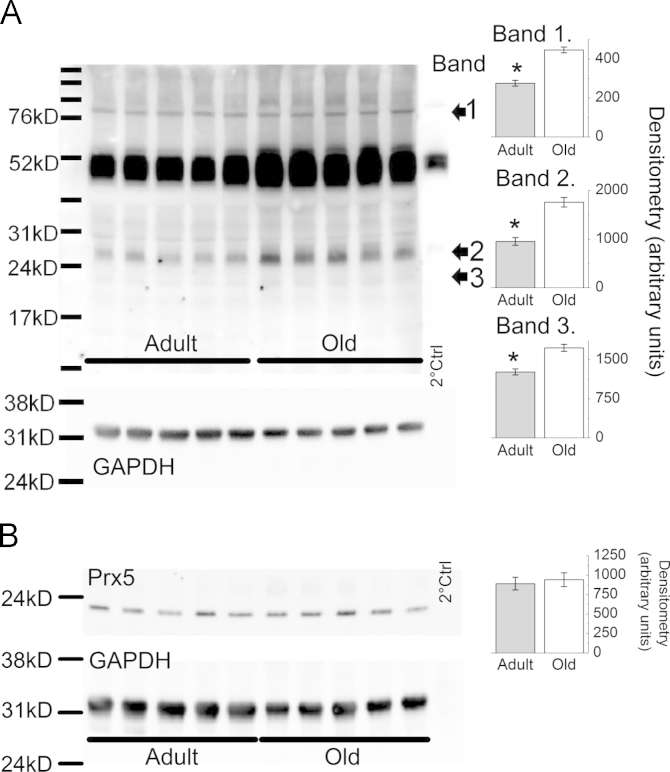
(A) Representative Western blot of 3-NT content of proteins from gastrocnemius muscles of adult and old mice (*n*=5, both groups). Densitometric analysis of the bands labeled 1–3 are shown on inset histograms. ^⁎^*P*<0.05 using Student’s *t* test). The GAPDH content from the same blot used as a loading control for the 3-NT blot is shown below. (B) Representative blot of peroxiredoxin V in gastrocnemius muscle from adult and old mice (*n*=5, both groups). Densitometry analysis (inset histogram) showed no differences between the groups. The GAPDH content as a loading control for the Prx5 blot is shown below.

Fig. 4A shows a Western blot probed for the 3-NT content of muscle proteins. Three bands were quantified by densitometry and analysed to compare muscles from adult and old mice. All of the 3 bands showed significantly (Student’s *t* test, *P*<0.05) greater 3-NT in the muscles from old mice. Previous studies have identified band 2, at 28 kDa, as carbonic anhydrase III (Vasilaki et al., 2007), but the identities of the other bands are not known. The heavy band at approximately 50 kDa was also present with the secondary antibody control and is a nonspecific artefact caused by the mouse IgG heavy chain. No differences in loading control (GAPDH) were seen. Previous studies have reported that Prx5 content of muscle is increased by exposure to peroxynitrite [8], but no differences were found between muscles from old compared with adult mice (Fig. 4B, Student’s *t* test, *P*>0.05). The loading control GAPDH showed no differences between samples from the different age groups.

## Discussion

The role of reactive oxygen and nitrogen species in skeletal muscle aging has received considerable attention, but whether muscle-derived NO is elevated or decreased with aging and how this influences the activities of superoxide has not previously been defined. The DAF-FM fluorescent probe permits analyses of NO in various cell types including skeletal muscle [29,32,33] and its use together with other ROS-sensitive probes in the current study has demonstrated an increase in NO in contracted skeletal muscle fibers from old mice in comparison with those from adult animals. Furthermore the presence of NO appeared to reduce the availability of superoxide in mitochondria in muscle fibers from both adult and old mice, but no age-related effect was seen.

All three NOS isoforms were found in the gastrocnemius muscle in common with previous published data [34,35]. Whole muscles were used for these analyses and hence it is not possible to draw any conclusions concerning any fiber type specificity of the NOS protein expression. Studies indicate that nNOS in skeletal muscle is located predominantly at the plasma membrane associated with the dystrophin glycoprotein complex [36,37], while eNOS is located to mitochondria [12,38]. The contraction-induced increase in NO observed in skeletal muscle has been predominantly attributed to nNOS activity [29,38]. Our previous studies have shown an interaction between NO and superoxide generated within skeletal muscle to form peroxynitrite [17,18], and this effect appears to be most predominant in mitochondria [31].

There is no current concensus on the changes in NOS enzymes and NO availability that occur during aging in skeletal muscle [23–27,39]. The data presented here showed no change in iNOS or nNOS content in muscle from old mice, but an increase in eNOS content with aging in skeletal muscle (Fig. 1). Since eNOS is predominantly located in mitochondria in skeletal muscle, this may reflect a specific change in eNOS content or could be influenced by a large change in mitochondria number. During aging fast muscle fiber types appear to be preferentially lost, fast fibers tend to contain less mitochondria than red slow muscle fibers, and a change in fiber types might account for some increase in eNOS expression , although reported changes in mitochondria number during aging appear insufficient to account for this ~100% increase in eNOS [40]. Song et al. [41] have reported no effect of aging (or exercise) on eNOS expression in rat soleus muscle, although they reported an increased eNOS expression with exercise in white gastrocnemius muscles.

Data showed that the NO content of fibers from old mice was increased following contractions in comparison with fibers from adult mice (Fig. 2). By comparison with the data on NOS proteins in muscle (Fig. 1) it is tempting to speculate that the increased NO availability was due to an increase in mitochondria-localised eNOS in fibers from old mice, but previous data indicate that nNOS was the predominant enzyme responsible for NO generation in muscles in response to contractile activity [29,38,42]. We have previously reported increased NO generation in muscle in response to passive stretching [43] and hypothesised that this was related to increased expression of iNOS in muscle from old mice. The current data do not allow us to fully answer the question relating to the likely source of increased NO seen in contracted muscle fibers from old mice.

Inhibition of NOS enzymes with L-NAME has previously been shown to decrease DAF-FM fluorescence, indicating decreased NO generation, in this specific isolated muscle fiber model [29] and in the current study was found to induce an increase in superoxide specifically within mitochondria (Fig. 3) as we have previously demonstrated [31]. The effect tended to be particularly marked in fibers from old mice, although fibers from adult mice also showed the same changes and the overall generation of superoxide within mitochondria or the cytosol of fibers from old mice was not significantly different from that seen from fibers of adult mice. Previous studies have examined the relationship between direct measurement of 2-hydroxyethidium formation to monitor superoxide activity using a HPLC-based approach and monitoring of fluorescence emissions from single FDB fibers following excitation at 405 nm [17]. Those data showed a similar pattern of changes in FDB fibers using both approaches and for convenience the fluorescence microscopy approach was used in the current studies [17]. The data obtained here therefore argue against an increased generation of superoxide from the mitochondrial electron transport chain playing a key role in aging processes as has been previously proposed [19–22], but it should be noted that the presence of relatively large amounts of SOD2 (MnSOD) in mitochondria will maintain superoxide levels at very low levels and that the activity of this enzyme is reported to be increased in muscle from old mice [44] and man [45]. Studies of the amount of hydrogen peroxide generated (the product of SOD-catalysed and spontaneous dismutation of superoxide) has generally indicated that this is increased from mitochondria isolated from old mice [40,46], suggesting that the flux of superoxide through mitochondria from old mice may be elevated rather than the concentration of superoxide increased.

The increased NO detected in muscle fibers and the increase in superoxide detected in the presence of the NOS inhibitor suggest that substantial peroxynitrite may be formed in muscle during contractile activity. Previous studies have examined the 3-NT content of a specific muscle protein, carbonic anhydrase III, and demonstrated this to be elevated following contractile activity in muscle from adult mice and in muscle from old mice at rest [8]. In the current study we showed a more generalised increase in the 3-NT content of muscle proteins. Three protein bands were detected that had elevated 3-NT, suggesting that muscle proteins from old mice has undergone substantial posttranslational changes due to reaction with peroxynitrite [14]. Tyrosine nitration can potentially cause detrimental changes in protein structure and function that may contribute to the aging muscle phenotype [47]. In previous studies of mice lacking SOD1, an increase in muscle peroxynitrite reactions was observed and this was associated with an increased content of Prx5. This protein has peroxynitrite reductase activity and has been found to be upregulated in conditions associated with increased peroxynitrite activity [48,49], but a similar increase in content was not observed in the current study of muscles from old mice (Fig. 4). An alternative explanation for change in superoxide detected in mitochondria of muscle fibers treated with the NOS inhibitor is that NO may interact with the electron transport chain to modify superoxide generation and this interaction would be removed by L-NAME treatment [50].

We have previously studied the processes of muscle loss in mice lacking SOD1 as a model with an accelerated aging phenotype that may provide mechanistic insight into the processes of muscle fiber loss and weakness that occurs in aging [15,51–53]. An increase in peroxynitrite activity can be predicted to occur in this model since the genetic defect leads to decreased dismutation of superoxide to hydrogen peroxide, thus increasing the relative amount of superoxide available to react with NO and form peroxynitrite. The finding of an increased NO content with increased an increased 3-NT content of muscle proteins is also apparent in old wild-type mice and therefore provides further support for the utility of the SOD1 null mouse model as a relevant model of skeletal muscle aging.

In conclusion, the data obtained are partly consistent with our hypothesis since they indicate that NO bioavailability is increased in muscle from old mice which showed a substantial age-related increase in NO levels in muscle fibers during contractile activity. This increase was associated with an increase in muscle eNOS content and muscle proteins from old mice had increased 3-NT content. Inhibition of NOS was also found to increase superoxide bioavailability in muscle mitochondria, although this effect was not age-related. Thus increased NO in muscles of old mice was associated with an increased 3-NT content and hence may potentially contribute to age-related degenerative changes in skeletal muscle.

## References

[1] Ryall J.G., Schertzer J.D., Lynch G.S. (2008). Cellular and molecular mechanisms underlying age-related skeletal muscle wasting and weakness. Biogerontology.

[2] Lexell J. (1995). Human aging, muscle mass, and fiber type composition. J. Gerontol. A Biol. Sci. Med. Sci.

[3] Porter M.M., Vandervoort A.A., Lexell J. (1995). Aging of human muscle: structure, function and adaptability. Scand. J. Med. Sci. Sports.

[4] Morley J.E. (2002). A fall is a major event in the life of an older person. J. Gerontol. A Biol. Sci. Med. Sci.

[5] Young A., Skelton D.A. (1994). Applied physiology of strength and power in old age. Int. J. Sports Med..

[6] Sastre J., Pallardó F.V., Viña J. (2012). The role of mitochondrial oxidative stress in aging. Free Radic. Biol. Med..

[7] Broome C.S., Kayani A.C., Palomero J., Dillmann W.H., Mestril R., Jackson M.J., McArdle A. (2006). Effect of lifelong overexpression of HSP70 in skeletal muscle on age-related oxidative stress and adaptation after non-damaging contractile activity. FASEB J.

[8] Vasilaki A., Simpson D., McArdle F., McLean L., Beynon R.J., Van Remmen H., Richardson A.G., McArdle A., Faulkner J.A., Jackson M.J. (2007). Formation of 3-nitrotyrosines in carbonic anhydrase III is a sensitive marker of oxidative stress in skeletal muscle. Proteomics Clin. Appl..

[9] Melov S., Ravenscroft J., Malik S., Gill M.S., Walker D.W., Clayton P.E., Wallace D.C., Malfroy B., Doctrow S.R., Lithgow G.J. (2000). Extension of life-span with superoxide dismutase/catalase mimetics. Science.

[10] Reid M,B., Shoji T., Moody M.R., Entman M.L. (1992). Reactive oxygen in skeletal muscle. II. Extracellular release of free radicals. J. Appl. Physiol..

[11] Balon T.W., Nadler J.L. (1994). Nitric oxide release is present from incubated skeletal muscle preparations. J. Appl. Physiol..

[12] Kobzik L., Reid M.B., Bredt D.S, Stamler J.S. (1994). Nitric oxide in skeletal muscle. Nature.

[13] Close G.L., Ashton T., McArdle A., Jackson M.J. (2005). Microdialysis studies of extracellular reactive oxygen species in skeletal muscle: factors influencing the reduction of cytochrome c and hydroxylation of salicylate. Free Radic. Biol. Med..

[14] Beckman J.S., Koppenol W.H. (1996). Nitric oxide, superoxide, and peroxynitrite: the good, the bad, and ugly. Am J. Physiol..

[15] Palomero J., Vasilaki A., Pye D., McArdle A., Jackson M.J. (2013). Aging increases the oxidation of dichlorohydrofluorescein in single isolated skeletal muscle fibers at rest, but not during contractions. Am J. Physiol. Regul. Integr. Comp. Physiol.

[16] Murrant C.L., Reid M.B. (2001). Detection of reactive oxygen and reactive nitrogen species in skeletal muscle. Microsc. Res. Tech..

[17] Sakellariou G.K., Pye D., Vasilaki A., Zibrik L., Palomero J., Kabayo T., McArdle F., Van Remmen H., Richardson A., Tidball J.G., McArdle A., Jackson M.J. (2011). Role of superoxide-nitric oxide interactions in the accelerated age-related loss of muscle mass in mice lacking Cu,Zn superoxide dismutase. Aging Cell.

[18] Pattwell D.M., McArdle A., Morgan J.E., Patridge T.A., Jackson M.J. (2004). Release of reactive oxygen and nitrogen species from contracting skeletal muscle cells. Free Radic. Biol. Med..

[19] Sawada M., Carlson J.C. (1987). Changes in superoxide radical and lipid peroxide formation in the rain, heart and liver during the lifetime of the rat. Mech. Ageing Dev..

[20] Sohal R.S., Svensson I., Sohal B.H., Brunk U.T. (1989). Superoxide anion radical production in different animal species. Mech. Ageing Dev..

[21] Ali S,S., Xiong C., Lucero J., Behrens M.M., Dugan L.L., Quick K.L. (2006). Gender differences in free radical homeostasis during aging: shorter-lived female C57BL6 mice have increased oxidative stress. Aging Cell.

[22] Sasaki T., Unno K., Tahara S., Shimada A., Chiba Y., Hoshino M., Kaneko T. (2008). Age-related increase of superoxide generation in the brains of mammals and birds. Aging Cell.

[23] Samengo G., Avik A., Fedor B., Whittaker D., Myung K.H., Wehling-Henricks M., Tidball J.G. (2012). Age-related loss of nitric oxide synthase in skeletal muscle causes reductions in calpain S-nitrosylation that increase myofibril degradation and sarcopenia. Aging Cell.

[24] Leiter J.R., Upadhaya R., Anderson J.E. (2012). Nitric oxide and voluntary exercise together promote quadriceps hypertrophy and increase vascular density in female 18-mo-old mice. Am. J. Physiol. Cell Physiol.

[25] Braga M., Sinha Hikim A.P., Datta S., Ferrini M.G., Brown D., Kovacheva E.L., Gonzalez-Cadavid N.F., Sinha-Hikim I. (2008). Involvement of oxidative stress and caspase 2-mediated intrinsic pathway signaling in age-related increase in muscle cell apoptosis in mice. Apoptosis.

[26] Ropelle E.R., Pauli J.R., Cintra D.E., da Silva A.S., De Souza C.T., Guadagnini D., Carvalho B.M., Caricilli A.M., Katashima C.K., Carvalho-Filho M.A., Hirabara S., Curi R., Velloso L.A., Saad M.J., Carvalheira J.B. (2013). Targeted disruption of inducible nitric oxide synthase protects against aging, S-nitrosation, and insulin resistance in muscle of male mice. Diabetes.

[27] Nyberg M., Blackwell J.R., Damsgaard R., Jones A.M., Hellsten Y., Mortensen S.P. (2012). Lifelong physical activity prevents an age-related reduction in arterial and skeletal muscle nitric oxide bioavailability in humans. J. Physiol..

[28] Shefer G., Yablonka-Reuveni Z. (2012). Isolation and culture of skeletal muscle myofibers as a means to analyze satellite cells. Methods Mol. Biol..

[29] Pye D., Palomero J., Kabayo T., Jackson M.J. (2007). Real-time measurement of nitric oxide in single mature mouse skeletal muscle fibers during contractions. J. Physiol..

[30] Kojima H., Urano Y., Kikuchi K., Higuchi T., Hirata Y., Nagano T. (1999). Fluorescent indicators for imaging nitric oxide production. Angew. Chem. Int. Ed. Engl..

[31] Pearson T., Kabayo T, Ng R., Chamberlain J., McArdle A., Jackson M.J. (2014). Skeletal muscle contractions induce acute changes in cytosolic superoxide, but slower responses in mitochondrial superoxide and cellular hydrogen peroxide. PLoS One.

[32] Wang D., Wang C., Wu X., Zheng W., Sandberg K., Ji H., Welch W.J., Wilcox C.S. (2014). Endothelial dysfunction and enhanced contractility in microvessels from ovariectomized rats: roles of oxidative stress and perivascular adipose tissue. Hypertension.

[33] Cortese-Krott M.M., Rodriguez-Mateos A., Kuhnle G.G., Brown G., Feelisch M, Kelm M.A. (2012). Multilevel analytical approach for detection and visualization of intracellular NO production and nitrosation events using diaminofluoresceins. Free Radic. Biol. Med..

[34] McConell G.K., Bradley S.J., Stephens T.J., Canny B.J., Kingwell B.A., Lee-Young R.S. (2007). Skeletal muscle nNOS mu protein content is increased by exercise training in humans. Am. J. Physiol. Regul. Integr. Comp. Physiol.

[35] Lau K.S., Grange R.W., Isotani E., Sarelius I.H., Kamm K.E., Huang P.L., Stull J.T. (2000). nNOS and eNOS modulate cGMP formation and vascular response in contracting fast-twitch skeletal muscle. Physiol. Genomics.

[36] Grozdanovic Z., Baumgarten H.G. (1999). Nitric oxide synthase in skeletal muscle fibers: a signaling component of the dystrophin-glycoprotein complex. Histol. Histopathol..

[37] Chang W.J., Iannaccone S.T., Lau K.S., Masters B.S., McCabe T.J., McMillan K., Padre R.C., Spencer M.J., Tidball J.G., Stull J.T. (1996). Neuronal nitric oxide synthase and dystrophin-deficient muscular dystrophy. Proc. Natl. Acad. Sci. USA.

[38] Hirschfield W., Moody M.R., O’Brien W.E., Gregg A.R., Bryan R.M., Reid M.B. (2000). Nitric oxide release and contractile properties of skeletal muscles from mice deficient in type III NOS. Am J. Physiol. Regul. Integr. Comp. Physiol.

[39] Richmonds C.R., Boonyapisit K., Kusner L.L., Kaminski H.J. (1999). Nitric oxide synthase in aging rat skeletal muscle. Mech. Ageing Dev..

[40] Pulliam D.A., Bhattacharya A., Van Remmen H. (2013). Mitochondrial dysfunction in aging and longevity: a causal or protective role?. Antioxid. Redox Signal..

[41] Song W., Kwak H.B., Kim J.H., Lawler J.M. (2009). Exercise training modulates the nitric oxide synthase profile in skeletal muscle from old rats. J. Gerontol. A Biol. Sci. Med. Sci.

[42] Tidball J.G., Lavergne E., Lau K.S., Spencer M.J, Wehling M. (1998). Mechanical loading regulates NOS expression and activity in developing and adult skeletal muscle. Am. J. Physiol. Cell Physiol.

[43] Palomero J., Pye D., Kabayo T., Jackson M.J. (2012). Effect of passive stretch on intracellular nitric oxide and superoxide activities in single skeletal muscle fibers: influence of ageing. Free Radic. Res..

[44] Vasilaki A., McArdle F., Iwanejko L.M., McArdle A. (2006). Adaptive responses of mouse skeletal muscle to contractile activity: the effect of age. Mech. Ageing Dev..

[45] Cobley J.N., Sakellariou G.K., Owens D.J., Murray S., Waldron S., Gregson W., Fraser W.D., Burniston J.G., Iwanejko L.A., McArdle A., Morton J.P., Jackson M.J, Close G.L. (2014). Lifelong training preserves some redox-regulated adaptive responses after an acute exercise stimulus in aged human skeletal muscle. Free Radic. Biol. Med..

[46] Vasilaki A., Mansouri A., Van Remmen H., van der Meulen J.H., Larkin L., Richardson A.G., McArdle A., Faulkner J.A., Jackson M.J. (2006). Free radical generation by skeletal muscle of adult and old mice: effect of contractile activity. Aging Cell.

[47] Radi R. (2013). Peroxynitrite, a stealthy biological oxidant. J. Biol. Chem..

[48] Dubuisson M., Vander Stricht D., Clippe A., Etienne F., Nauser T., Kissner R., Koppenol W.H., Rees J.F., Knoops B. (2004). Human peroxiredoxin 5 is a peroxynitrite reductase. FEBS Lett..

[49] Trujillo M., Ferrer-Sueta G., Radi R. (2008). Peroxynitrite detoxification and its biologic implications. Antioxid. Redox Signal..

[50] Erusalimsky J.D., Moncada S. (2007). Nitric oxide and mitochondrial signaling: from physiology to pathophysiology. Arterioscler. Thromb. Vasc. Biol..

[51] Muller F.L., Song W., Liu Y., Chaudhuri A., Pieke-Dahl S., Strong R., Huang T.T., Epstein C.J., Roberts L.J., Csete M., Faulkner J.A., Van Remmen H. (2006). Absence of CuZn superoxide dismutase leads to elevated oxidative stress and acceleration of age-dependent skeletal muscle atrophy. Free Radic. Biol. Med..

[52] Vasilaki A, van der Meulen J.H., Larkin L., Harrison D.C., Pearson T., Van Remmen H., Richardson A., Brooks S.V., Jackson M.J., McArdle A. (2010). The age-related failure of adaptive responses to contractile activity in skeletal muscle is mimicked in young mice by deletion of Cu,Zn superoxide dismutase. Aging Cell.

[53] Sakellariou G.K., Davis C.S., Shi Y., Ivannikov M.V., Zhang Y., Vasilaki A., Macleod G.T., Richardson A., Van Remmen H., Jackson M.J., McArdle A., Brooks S.V. (2014). Neuron-specific expression of CuZnSOD prevents the loss of muscle mass and function that occurs in homozygous CuZnSOD-knockout mice. FASEB J.

